# C1q Binding Ability for Prior Risk Assessment of Acute Antibody-Mediated Rejection in ABO-Incompatible Kidney Transplantation

**DOI:** 10.3389/ti.2024.13407

**Published:** 2024-10-15

**Authors:** Yuko Miwa, Kenta Iwasaki, Kenta Murotani, Manabu Okada, Takaharu Nagasaka, Yoshihiko Watarai, Asami Takeda, Masato Shizuku, Satoshi Ashimine, Kohei Ishiyama, Shoichi Maruyama, Takaaki Kobayashi

**Affiliations:** ^1^ Department of Kidney Disease and Transplant Immunology, Aichi Medical University School of Medicine, Nagakute, Japan; ^2^ Biostatistics Center, Kurume University, Kurume, Japan; ^3^ Department of Transplant Surgery, Japanese Red Cross Aichi Medical Center Nagoya Daini Hospital, Nagoya, Japan; ^4^ Department of Transplant Surgery, Toyohashi Municipal Hospital, Toyohashi, Japan; ^5^ Department of Nephrology, Masuko Memorial Hospital, Nagoya, Japan; ^6^ Department of Renal Transplant Surgery, Aichi Medical University School of Medicine, Nagakute, Japan; ^7^ Department of Nephrology, Nagoya University Graduate School of Medicine, Nagoya, Japan

**Keywords:** ABO-incompatible kidney transplantation, acute antibody-mediated rejection, A/B antigen expression levels of donor specimens, IgG subclasses, C1q binding ability

## Abstract

In ABO blood group incompatible kidney transplantation (ABO-I), potential issues on acute antibody-mediated rejection (ABMR) remain to be solved. This study aimed to assess the risk factors of acute ABMR using recipient- or donor-derived specimens. Quantitative analysis of A/B antigen expression was conducted in 104 donor kidney tissues (Kt), platelets (Plt), and red blood cells (RBC) by immunohistochemical staining or flow cytometry (FCM). ABO-I pre-transplant recipient serum samples (ABMR = 12, non-ABMR = 27) were extracted by propensity score matching. Anti-A antibody titers of IgM, IgG and IgG subclasses, and C1q binding ability (%) on antibody were measured using RBC-FCM. No association was observed between ABMR and A/B antigen expression levels in donor’s Plt, RBC, or Kt. In recipient’s sample, C1q-IgG binding ability was significantly higher in the ABMR group than in the non-ABMR group (C1q−IgG: 9.04% vs. 5.93% *p* = 0.049). Neither the A/B antigen expression level in donors (grafts) nor anti-blood group IgG/IgM antibodies in recipient sera before desensitization seemed to influence ABMR incidence in ABO-I. In contrast, C1q-IgG binding ability could be a potential predictor for ABMR in ABO-I.

## Graphical Abstract

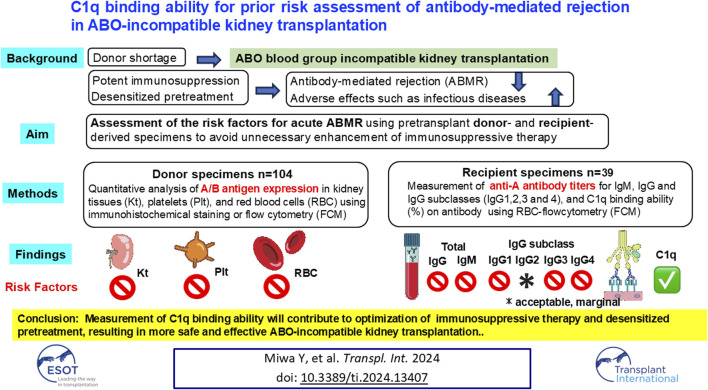

## Introduction

Effective desensitization therapy has improved the outcomes of ABO-incompatible (ABO-I) kidney transplantation [1, 2]. However, the graft survival rate of ABO-I is slightly inferior to ABO-Identical/compatible kidney transplantation (ABO-Id/C) [3, 4]. This may be due not to acute antibody-mediated rejection (ABMR) but adverse effects such as infectious diseases [5]. The recent SARS-COV-2 pandemic has caused fear of infection in immunosuppressed transplant patients [6, 7]. Furthermore, patients under rituximab (RIT) treatment showed low vaccine efficacy [8]. Therefore, unnecessary desensitization therapy should be avoided. Optimization of immunosuppressive therapy (IST) including desensitization by risk stratification of acute ABMR (i.e., reduction of desensitization regimen for patients with a low risk) may further improve outcomes in ABO-I.

The intensity of the antigen-antibody reaction is defined by the density of antigen expression and the amount of antibody. Determining the antigen expression in vascular endothelial cells (EC) of donor grafts before transplantation could provide important information on donor risk factors. However, since the kidney tissues (Kt) of donors are not commonly available before transplantation, platelets (Plt) and red blood cells (RBC) in the peripheral blood, and which express blood group A/B antigens [9–12], were examined to test whether their expression levels correlate with the amount of A/B antigen in the graft’s EC. Although the carbohydrate binding protein [13] or carbohydrate chain of a glycan precursor [14, 15] properties of A/B antigens seem to differ between RBC and EC, it would be important to know whether the A/B antigen expression levels in RBC or Plt can reflect those in EC. Currently, measuring the anti-A/B antibody titer in the recipient serum by hemagglutination is widely used as a main pre-test in ABO-I, but anti-A/B antibody titer alone may be insufficient in clinical settings [16, 17].

Another risk factor, this time in recipients, is related to a difference in complement activation ability between IgG subclasses: both IgG1 and IgG3 have higher ability than IgG2 or IgG4 [18, 19]. Furthermore, the IgG1, IgG2, IgG3, and IgG4 distribution in peripheral blood differs from person to person [14, 20]. However, whether the patterns of IgG subclasses in the recipient’s pre-transplant blood can be a risk factor of acute ABMR in ABO-I remains unknown. In contrast, in HLA (Human Leukocyte antigen) -incompatible kidney transplantation (HLA-I), there are reports on the value of IgG subclass post-transplant measurement in recipients as a prognostic marker [21–23]. C1q, the first component of the complement activation through the classical pathway, binding ability to donor specific HLA antibody (DSA) has been associated with ABMR and graft loss [24, 25], whereas the correlation between ABMR incidence in ABO-I and C1q binding ability to anti-A/B antibody has not been reported yet.

In this study, we examined whether A/B antigen expression in the donor (Kt, RBC, and Plt) and C1q binding ability against donor RBC, and anti-A antibodies in recipient sera could predict ABMR in ABO-I.

## Materials and Methods

### Study Design and Patients

#### [Donor Patients] Case Control Study 1

**FIGURE 1:**
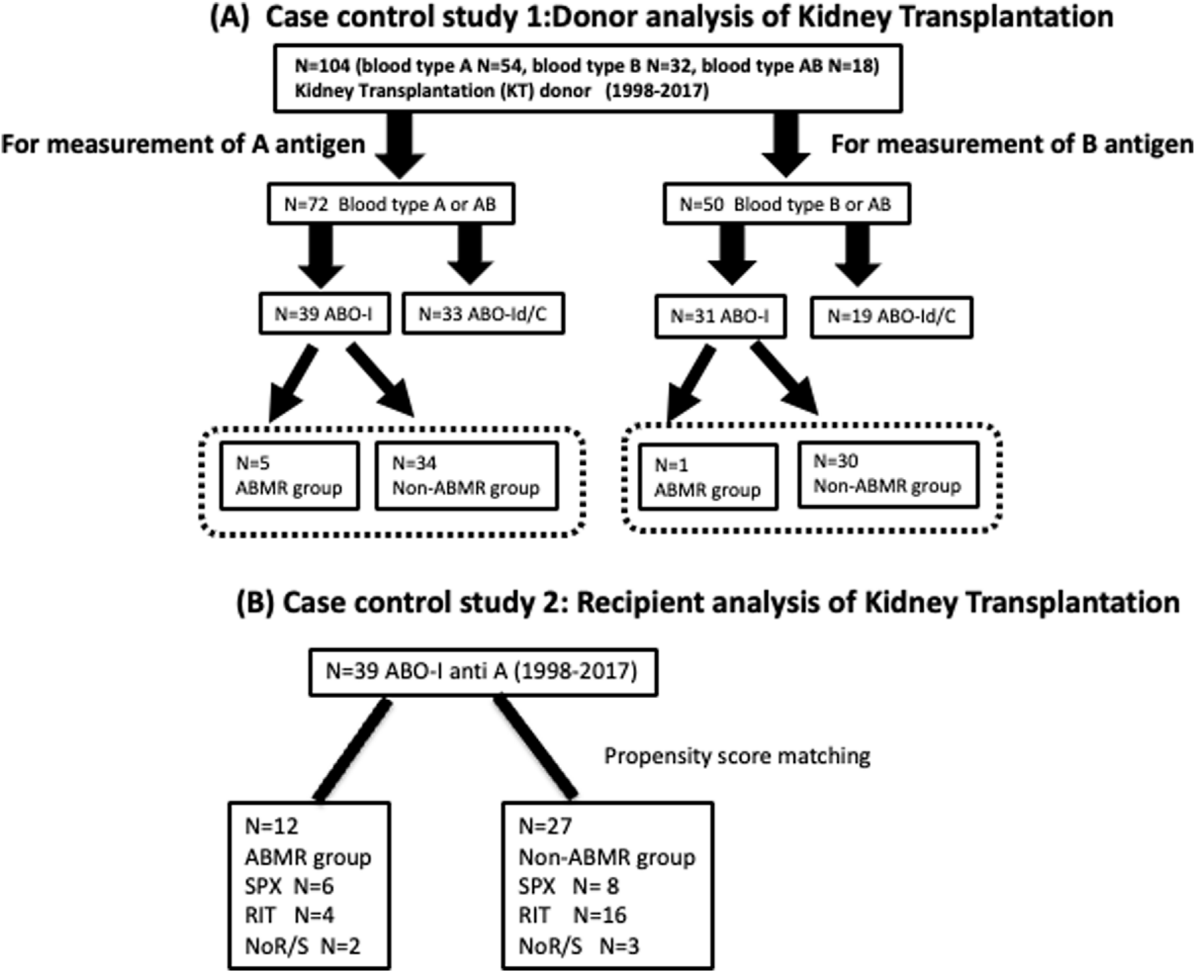
Flow chart of patient selection **(A)** Case control study 1: Donor analysis of kidney transplantation Plt, RBC, and Kt were collected from 104 ABO-I and ABO-Id/C donors to measure A/B antigen expression. Then, in ABO-I, we compared whether there was a difference in A antigen expression between the ABMR and non-ABMR groups. Plt, platelets; RBC, red blood cells; Kt, kidney tissues; ABO-I, ABO-incompatible kidney transplantation; ABO-Id/C, ABO-identical or compatible kidney transplantation. **(B)** Case control study 2: Recipient analysis of kidney transplantation. The background of anti-A 39 patients who underwent ABO-I at the Japanese Red Cross Aichi Medical Center Nagoya Daini Hospital between 1998 and 2017 were compared between ABMR and non-ABMR groups. RIT, rituximab; SPX, splenectomy; NoR/S, neither rituximab nor splenectomy.

Kidney grafts from 104 living donors (A group: n = 54, B group: n = 32, AB group: n = 18) were transplanted at the Japanese Red Cross Aichi Medical Center Nagoya Daini Hospital, between 1998 and 2017. Among 72 patients expressing A antigen in the grafts, 39 patients and 33 patients were ABO-I and ABO-Id/C, respectively. Five of the 39 ABO-I had ABMR. Similarly, only one in 30 ABO-I expressing blood group B in the graft showed acute ABMR (Figure 1A).

#### [Recipient Patients] Case Control Study 2

The backgrounds of 42 patients with blood group A mismatch who underwent ABO-I at the Japanese Red Cross Aichi Medical Center Nagoya Daini Hospital between 1998 and 2017 were compared after classification into the ABMR and non-ABMR groups (Figure 1B). Patients in the non-ABMR group were extracted based on propensity score matching; there was no significant difference in age, sex, blood group, desensitization therapy, and maintenance IST of recipient and donor patients. Patient characteristics are summarized in Table 1. The study was performed in accordance with the guidelines of the Declaration of Helsinki, after approval from the hospital’s institutional ethical committee of Aichi Medical University School of Medicine (authorization number 15-092, 15-H072).

**TABLE 1:** Characteristics of the anti A patients of ABO-I.

	ABO-I ABMR (n = 12)	ABO-I non-ABMR (n = 27)	P-value
Male, n (%)	6 (50.0)	18 (66.7)	0.323
Age, y.o, median (range)	46 (19–76)	52 (22–71)	0.268
Donor age, y.o, median (range)	59 (44–74)	62 (43–82)	0.277
ABO blood type of donor → Recipient			
*A→O, n (%)*	10 (83.3)	16 (59.3)	0.141
*AB→O, n (%)*	0	2 (7.4)	0.333
*A→B, n (%)*	2 (16.7)	6 (22.2)	0.692
*AB→B, n (%)*	0	3 (11.1)	0.229
Desensitization therapy			
Splenectomy (SPX), n (%)	6 (50.0)	8 (29.6)	0.221
Rituximab (RIT), n (%)	4 (33.3)	17 (59.3)	0.135
SPX (−), RIT (−) (%)	2 (16.7)	3 (11.1)	0.632
HLA antibody			
Anti HLA sesitized recipients (n,%)	0	0	—
*de novo* DSA (n,%)	0	4 (14.8)	0.159
Maintenance Immunosuppression			
*Cyclosporine A, n (%)*	10 (83.3)	22 (81.5)	0.889
Tacrolimus, n (%)	2 (16.7)	5 (18.5)	0.889

ABO-I, ABO blood group incompatible kidney transplantation; ABMR, Antibody-mediated rejection.

*P* < 0.05.

### Desensitization Protocol

ABO-I recipients were pretreated with mycophenolate mofetil (MMF) from day −14, double-filtration plasmapheresis (DFPP) and either splenectomy (SPX), rituximab (RIT) (200 mg/body; twice; days −14 and −1, available from 2008), or neither (due to low anti-A/B antibody titers). Preoperative DFPP was routinely performed four times (days −6, −4, −2, and −1) in RIT or SPX and twice (days −2 and −1) in NoR/S.

### Immunosuppression Protocol

All transplant recipients received 500 mg methylprednisolone intravenously before graft reperfusion and 20 mg of basiliximab intravenously on days 0 and 4. The immunosuppressive regimen consisted of a calcineurin inhibitor (cyclosporine or tacrolimus), an antimetabolite (MMF or mizoribine) or mammalian target of rapamycin inhibitor (everolimus, available from 2008), and prednisolone. The dosage of all oral immunosuppressive medications, except prednisolone, was strictly adjusted according to pharmacokinetics (AUC 0–4 h or trough level). Cyclophosphamide was used as an antimetabolite only in case of SPX.

### ABMR Diagnosis

In this study, recipients with preformed DSA were not extracted. Whenever rejection was clinically suspected, an episodic biopsy was performed. The diagnosis of rejection was made by a pathologist at the Japanese Red Cross Aichi Medical Center Nagoya Daini Hospital. If no anti-donor HLA Abs were detected at the time of rejection, the diagnosis of ABMR due to anti-A or anti-B Abs was made using the pathology findings of ABMR (Banff 1997, 2001, 2005, 2007, 2013, 2017) during the study period 1998-2017.

### Immunohistochemical Staining of Kt

Donor renal tissue of 1-h biopsy after transplantation was formalin-fixed and embedded in paraffin. Staining for blood group A and B antigen was performed on 1 µm thick paraffin embedded sections. After deparaffinization, sections were incubated with a monoclonal mouse IgM anti-A antibody (clone MH04,3D3; Ortho Clinical Diagnostics, Tokyo, Japan) and a monoclonal mouse IgM anti-B antibody (clone NE11.19,5A5,3D4; Ortho Clinical Diagnostics) as primary antibodies. Next, sections were incubated with Dako Envision detection System (DAKO, Glostrup, Denmark) as second antibody. Peroxidase activity was visualized by staining with a 3,3′-Diaminobenzidine, tetrahydrochloride (DAB) solution. Immunostained slides were scanned in a virtual slide microscopy (VS120, Olympus, Tokyo, Japan). In this study, the DAB stain area of A/B antigens, was measured using the image analysis software Tissuemorph DP (Visiopharm, Hoersholm, Denmark). A/B antigen expression was analyzed in three selected renal glomeruli; Tissuemorph DP shows the area of DAB stain in green, the nuclei in blue, and a region of interest (ROI) around blue dotted line. The index of A/B antigen immunopositivity was the ratio of the total DAB stain area and total ROI area (Max DAB/ROI value; Supplementary Figure S1) [26].

### Flow Cytometry Analysis of Blood Type A, B Antigen Expression on Plt and RBC

Platelet-rich plasma (PRP) was prepared by centrifugation of anti-coagulated whole blood in acid-citrate-dextrose (ACD) tube at 250 g for 15 min. Then, the PRP was diluted three times with 20% ACD in Plt buffer (0.14 M NaCl, 5 mM KCl, 1 mM MgSO4, and 10 mM HEPES, pH 7.4), and centrifuged at 750 *g* for 2.5 min to form platelet pellets. Plt were stabilized by fixation in paraformaldehyde at a final concentration of 1%. RBC was collected by centrifugation at 1,000 g from citric acid-treated blood and washed twice with PBS (−) containing 0.2% bovine serum albumin and 0.1% NaN_3_ (wash buffer). Then, they were incubated with 3 mg/mL dimethyl suberimidate dihydrochroride (DMS) in 0.1 M Na_2_CO_3_ containing 0.15 M NaCl and 0.1 mM EDTA at 37°C for 20 min to prevent agglutination. DMS-treated RBC were washed with wash buffer twice and suspended in wash buffer at 1% concentration. For the detection of blood group A/B antigen in Plt and RBC using flow cytometry (FACSCanto II, Becton Dickinson, San Jose, CA, United States), 4.0 ×10^6^ Plt and 4.5 × 10^5^ RBC were incubated with monoclonal mouse IgM anti-A or B antibody (Ortho Clinical Diagnostic) for 20 min at room temperature. Fluorescein (FITC)-labeled goat anti-mouse IgM (American Qualex Antibodies, San Clemente, CA) was used as secondary antibody. A/B antigen expression levels were analyzed by the mean fluorescence intensity (MFI).

### Detection of Anti-A IgG, IgM, and IgG Antibody Titers in Patient Serum

For the detection of anti-A antibody titer in patient pre-treatment serum using RBC flow cytometry, 30 µL of 1 × 10^7^/mL DMS-treated RBCs and 15 µL of heat-inactivated patient serum were incubated in 96-well plates for 20 min at room temperature. After three washes with 0.1% BSA in PBS (−), RBC were incubated with a diluted secondary antibody, either FITC-labeled rabbit anti-human IgG, IgM (DAKO) or R-phycoerythrin (R-PE)-labeled mouse IgG1, IgG2, IgG3, IgG4 (SouthernBiotech, Birmingham, AL, United States). The stained RBC were analyzed using high-throughput flow cytometry (FACS Canto II High Throughput Sampler option, Becton Dickinson), which allows simultaneous testing of large patient’s samples in 96-well plates. The anti-A antibody isohemagglutinin antibody titers for IgG and IgM were serially measured as previously reported [27].

### Detection of Complement C1q (C1q−IgG and C1q−IgG+IgM) Binding Ability in Patient Serum

To degrade IgM antibodies, heat-inactivated patient serum was incubated with 5 mM dithiothreitol (DTT) at 37°C for 30 min. At first 30 µL of 1 × 10^7^/mL DMS-treated RBC and 15 µL of patient serum (DTT treated or non-treated) were incubated for 20 min at room temperature. After three washes with 0.1% BSA in PBS (−), RBC were incubated with 5 µL of complement component C1q from human serum (Sigma-Aldrich, St Louis, MO, United States) in PBS (−) at room temperature for 20 min. Then, after adding 50 µL of ×20 diluted FITC-labeled rabbit polyclonal anti-human C1q antibody (ab4223; Abcam plc, Cambridge, United Kingdom), RBC were incubated at room temperature for 20 min. After washing RBC twice with 0.1% BSA in PBS (−), RBC were measured using flow cytometry (FACS Canto II, Becton Dickinson). To assess C1q binding ability, RBC reacted with C1q; secondary antibody were used only as negative controls and threshold lines were drawn at 3% C1q binding ability of the AB blood type serum and compared in terms of positivity rate (%).

### Statistical Analysis

The variability of groups with different units was expressed by the coefficient of variation (CV). The Mann–Whitney U test was used to compare two groups of continuous variables. Medians with a 25th and 75th percentile were calcurated. The cut-off value was determined by receiver operator characteristic curve (ROC) analysis using Youden index. Moreover, Fisher’s exact test in a 2 × 2 contingency table was used to compare categorical data between groups. P values < 0.05 were considered statistically significant. Statistical analyses were performed using GraphPad Prism ver 5.03 and JMP ver 13.2.

## Results

### Individual Differences in Blood Type A/B Antigen Expression in Donors’ Plt, RBC, and Kt

**FIGURE 2:**
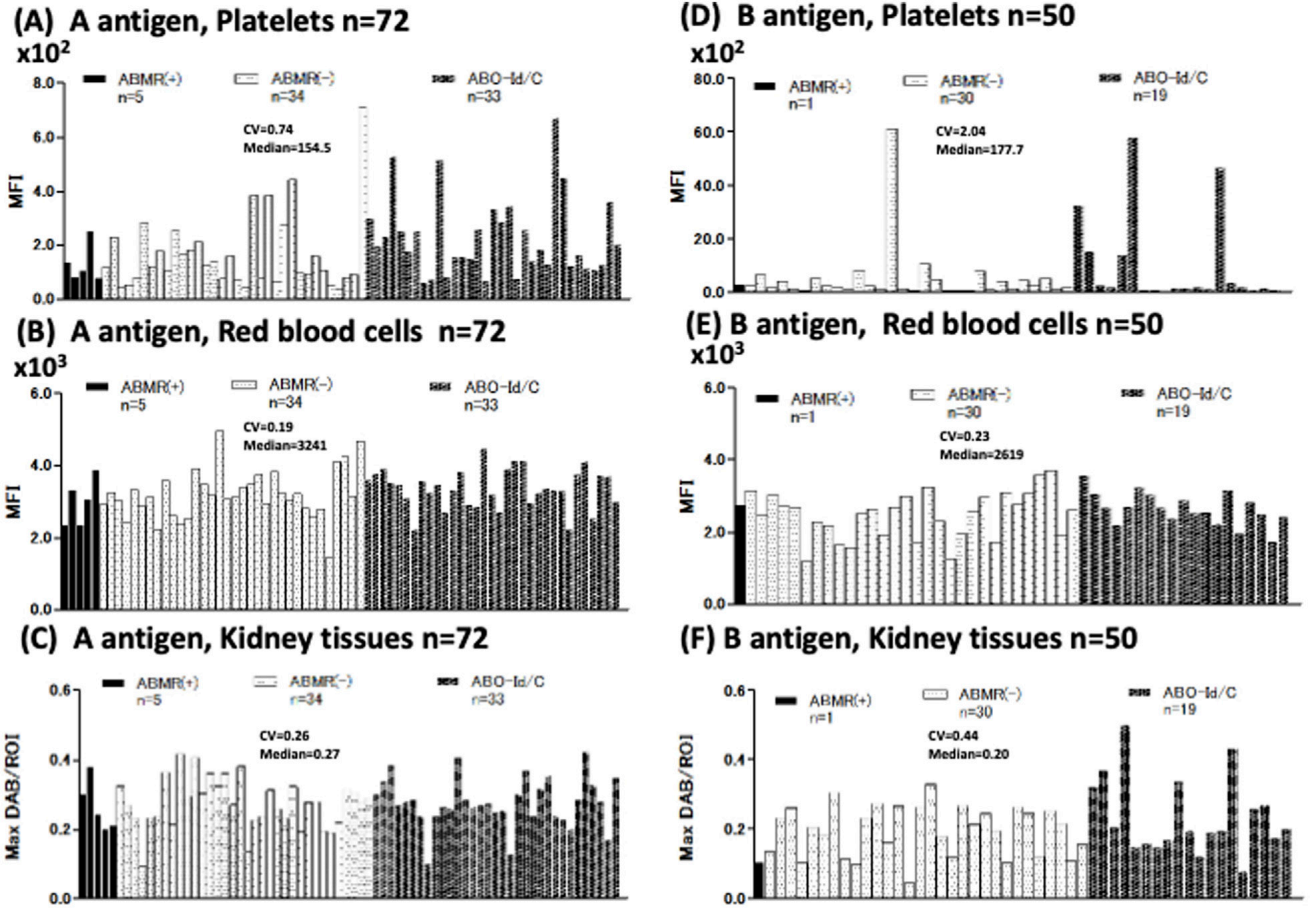
Individual differences in blood group A/B antigen expression in platelets (Plt), red blood cell (RBC), and kidney tissue (Kt). Blood group A antigen expression of Plt, RBC, and Kt **(A–C)** Antigen A expression of 72 donors [ABMR(+) n = 5, ABMR(−) n = 34, ABO-Id/C n = 33]. Coefficient of variation (CV), index for comparing individual differences. Individual differences in Plt’s antigen A expression were higher than in RBC and Kt (Plt, RBC, and Kt CV = 0.74, 0.19, and 0.26, respectively). B antigen expression of Plt, RBC, and Kt **(D–F)**. B antigen expression of 50 donors [ABMR(+) n = 1, ABMR(−) n = 30, ABO-Id/C n = 19]. Individual differences in Plt’s B antigen expression were strikingly higher than in RBC and Kt (Plt, RBC, and Kt CV were 2.04, 0.23, and 0.44, respectively).

We measured A/B antigen expression levels in the Plt, RBC, and Kt of 104 donors (Figure 1A), A antigen: n = 72 [ABMR(+) n = 5, ABMR(−) n = 34, ABO-Id/C n = 33], B antigen: n = 50 [ABMR(+) n = 1, ABMR(−) n = 30, ABO-Id/C n = 19] of donor patients (Figure 2). The inter-individual differences in both A and B antigen in Plt were larger than those in RBC and Kt [CV; 0.74 (Plt) vs. 0.19 (RBC) and 0.26 (Kt) in A antigen, 2.04 (Plt) vs. 0.23 (RBC) and 0.44 (Kt) in B antigen]. No correlation in A/B antigen expression levels was observed between Plt, RBC, and Kt (Figure 2).

### Expression Levels of Blood Type A Antigen of Plt, RBC, and Kt in ABMR and Non-ABMR Groups

**FIGURE 3:**
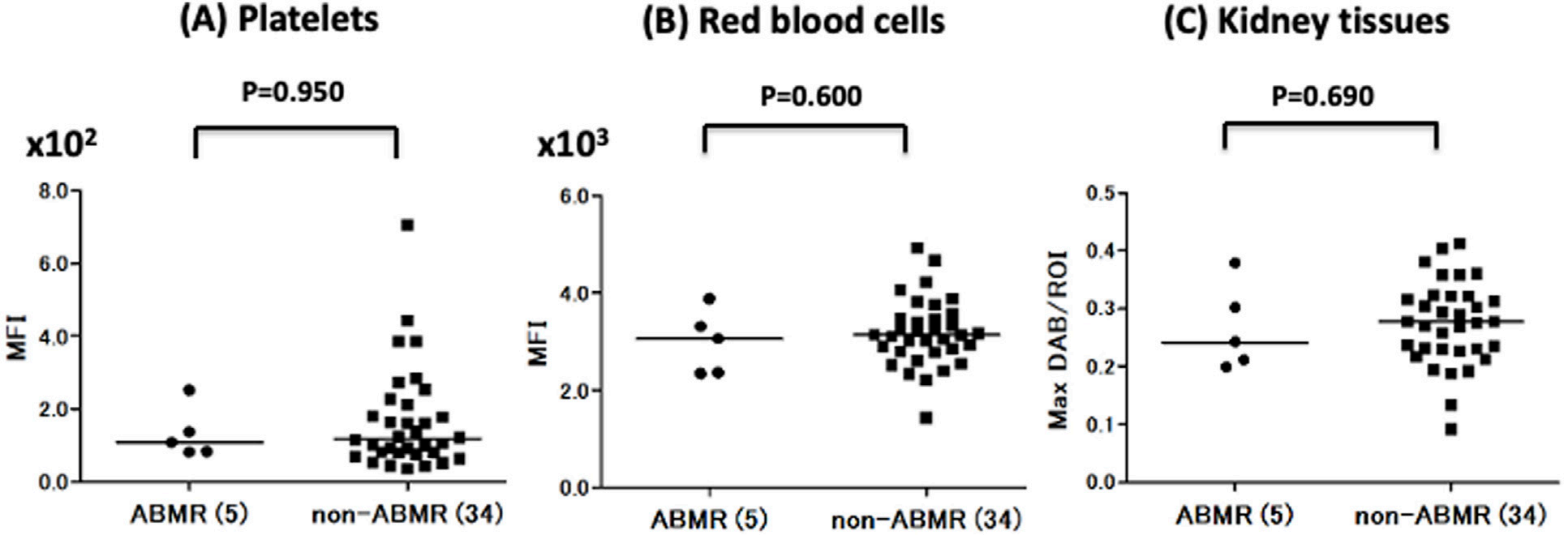
Comparison of Plt, RBC, and Kt’s blood group A antigen expression levels in the antibody-mediated rejection (ABMR) and non-ABMR groups. There were no significant differences in antigen A expression in Plt **(A)**, RBC **(B)**, and Kt **(C)** between the ABMR group and non-ABMR group.

Next, we compared A antigen expression in the Plt, RBC, and Kt of ABO-I donors between ABMR and non-ABMR groups. No significant difference in A antigen expression levels was observed between groups (Figure 3). Regarding B antigen expression, although statistical analysis could not be performed because of the very small number of patients with ABMR, no increasing tendency was observed in B antigen expression levels in the ABMR group (Figures 2D–F).

### Anti-A Total IgG and IgM Titers in ABMR and Non-ABMR Groups

**FIGURE 4:**
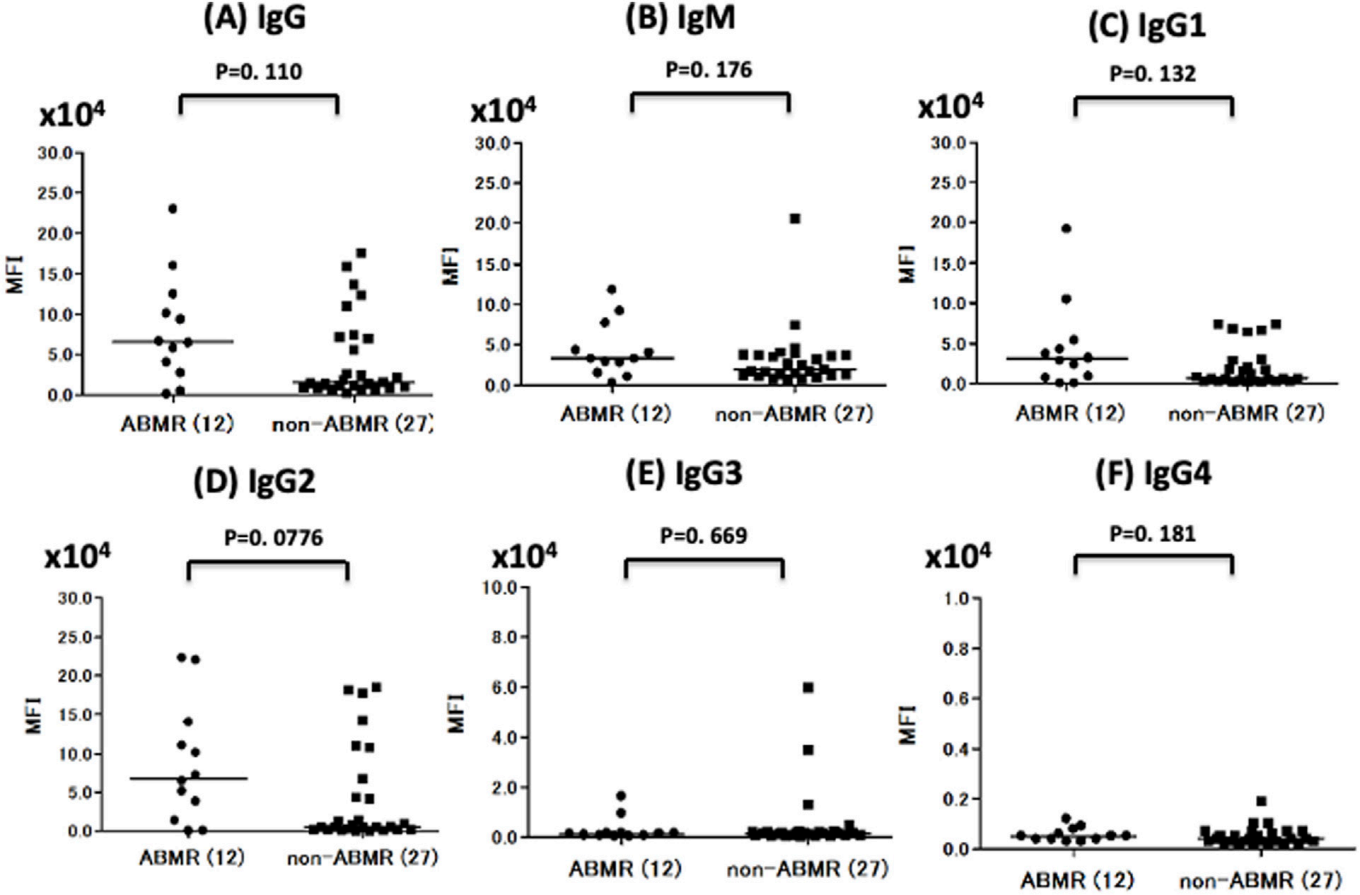
Comparison of anti-A IgG, IgM, and IgG subclass titer in ABMR and non-ABMR groups. The anti-A antibody total IgG median titer (MFI) was higher in the ABMR group than in the non-ABMR (*p* = 0.110). **(A)** The anti-A antibody total IgM MFI was higher in the ABMR group than in the non-ABMR (*p* = 0.175). **(B)** In anti-A, IgG1 and IgG2 had no significant difference between ABMR group and non-ABMR group. [IgG1: *p* = 0.131, IgG2: *p* = 0.077, IgG3: *p* = 0.669, IgG4 = 0.180; **(C–F)**].

Anti-A antibody median total IgG titers were higher in the ABMR group than in the non-ABMR [MFI: 6.59 × 10^4^ (25th–75th percentile, 3.08 × 10^4^–11.9 × 10^4^) vs. MFI: 1.53 × 10^4^ (25th–75th percentile, 1.01 × 10^4^–7.13 × 10^4^; *p* = 0.110)], as were anti-A antibody total IgM median titers [MFI: 3.35 × 10^4^ (25th–75th percentile, 1.91 × 10^4^–6.91 × 10^4^) vs. MFI: 1.96 × 10^4^ (25th–75th percentile, 1.15 × 10^4^–3.74 × 10^4^; *p* = 0.175)] (Figure 4; Table 2). MFI values were normalized to those obtained in normal control serum. The cut-off values were calculated from ROC analysis [anti-A IgG: 2.76 × 10^4^, which is a hemagglutination test equivalent to 64 times, area under the curve (AUC) = 0.664, IgM: 2.89 × 10^4^, which is the hemagglutination test equivalent to 32 times, AUC = 0.639] (Table 2; Supplementary Figure S3). Statistical analysis of anti-B titers was not possible due to the small number of ABMR patients.

**TABLE 2:** Comparison of the patients with anti A IgG, IgM, IgG subclass and C1q titer of ABMR and non-ABMR group in ABO-I.

	Median (25th and 75th percentile)		Mann-Whitney U-test	ROC curve (Receiver Operator Characteristic Curve) analysis	
	ABMR group (n=12)	non-ABMR group (n=27)	*P* value	cut off (IHT)	AUC
IgG	6.59 × 10^4^ (3.08 × 10^4^–11.9 × 10^4^)	1.53 × 10^4^ (1.01 × 10^4^–7.13 × 10^4^)	0.110	2.76 × 10^4^ (×64)	0.664
IgM	3.35 × 10^4^ (1.91 × 10^4^–6.91 × 10^4^)	1.96 × 10^4^ (1.15 × 10^4^–3.74 × 10^4^)	0.175	2.89 × 10^4^ (×32)	0.639
IgG1	3.07 × 10^4^ (0.81 × 10^4^–5.12 × 10^4^)	0.67 × 10^4^ (0.24 × 10^4^–2.89 × 10^4^)	0.131	2.40 × 10^4^	0.654
IgG2	6.85 × 10^4^ (2.02 × 10^4^–13.4 × 10^4^)	0.54 × 10^4^ (0.19 × 10^4^–6.77 × 10^4^)	0.077	1.39 × 10^4^	0.679
IgG3	0.13 × 10^4^ (0.07 × 10^4^–0.16 × 10^4^)	0.15 × 10^4^ (0.07 × 10^4^–0.20 × 10^4^)	0.669	0.16 × 10^4^	0.537
IgG4	0.05 × 10^4^ (0.04 × 10^4^–0.08 × 10^4^)	0.04 × 10^4^ (0.03 × 10^4^–0.07 × 10^4^)	0.180	0.03 × 10^4^	0.639
C1q-IgG	9.04% (7.63–26.7)	5.93% (4.48–10.3)	0.049	7.47%	0.701
C1q-IgG+IgM	37.4% (5.48–80.5)	9.70% (2.00–57.1)	0.120	26.6%	0.659

ABMR, Antibody-mediated rejection; IHT, Isohemagglutinin titer; AUC, Aria under the ROC curve.

*P* < 0.05.

### Anti-A IgG Subclass Distribution in ABMR and Non-ABMR Groups

The anti-A antibody IgG1, IgG2, IgG3, and IgG4 levels were not significantly higher in the ABMR group than in the non-ABMR group [MFI: 3.07 × 10^4^ (25th–75th percentile, 0.81 × 10^4^–5.12 × 10^4^) vs. MFI: 0.67 × 10^4^ (25th–75th percentile, 0.24 × 10^4^–2.89 × 10^4^; *p* = 0.131 in IgG1], [MFI: 6.85 × 10^4^ (25th–75th percentile, 2.02 × 10^4^–13.4 × 10^4^) vs. MFI: 0.54 × 10^4^ (25th–75th percentile, 0.19 × 10^4^–6.77 × 10^4^), *p* = 0.077 in IgG2], [MFI: 0.13 × 10^4^ (25th–75th percentile, 0.07 × 10^4^–0.16 × 10^4^) vs. MFI: 0.15 × 10^4^ (25th–75th percentile, 0.07 × 10^4^–0.20 × 10^4^), *p* = 0.669 in IgG3], [MFI: 0.05 × 10^4^ (25th–75th percentile, 0.04 × 10^4^–0.09 × 10^4^) vs. MFI: 0.04 × 10^4^ (25th–75th percentile, 0.04 × 10^4^–0.08 × 10^4^), *p* = 0.180 in IgG4], (Figure 4; Table 2). The MFI cut-off values were calculated from ROC analysis (IgG1: 2.40 × 10^4^, AUC = 0.654, IgG2: 1.39 × 10^4^, AUC = 0.679, IgG3: 0.16 × 10^4^, AUC = 0.537, IgG4: 0.03 × 10^4^, AUC = 0.639: Table 2).

### C1q Binding Ability to Anti-A Antibody in ABMR and Non-ABMR Groups

**FIGURE 5:**
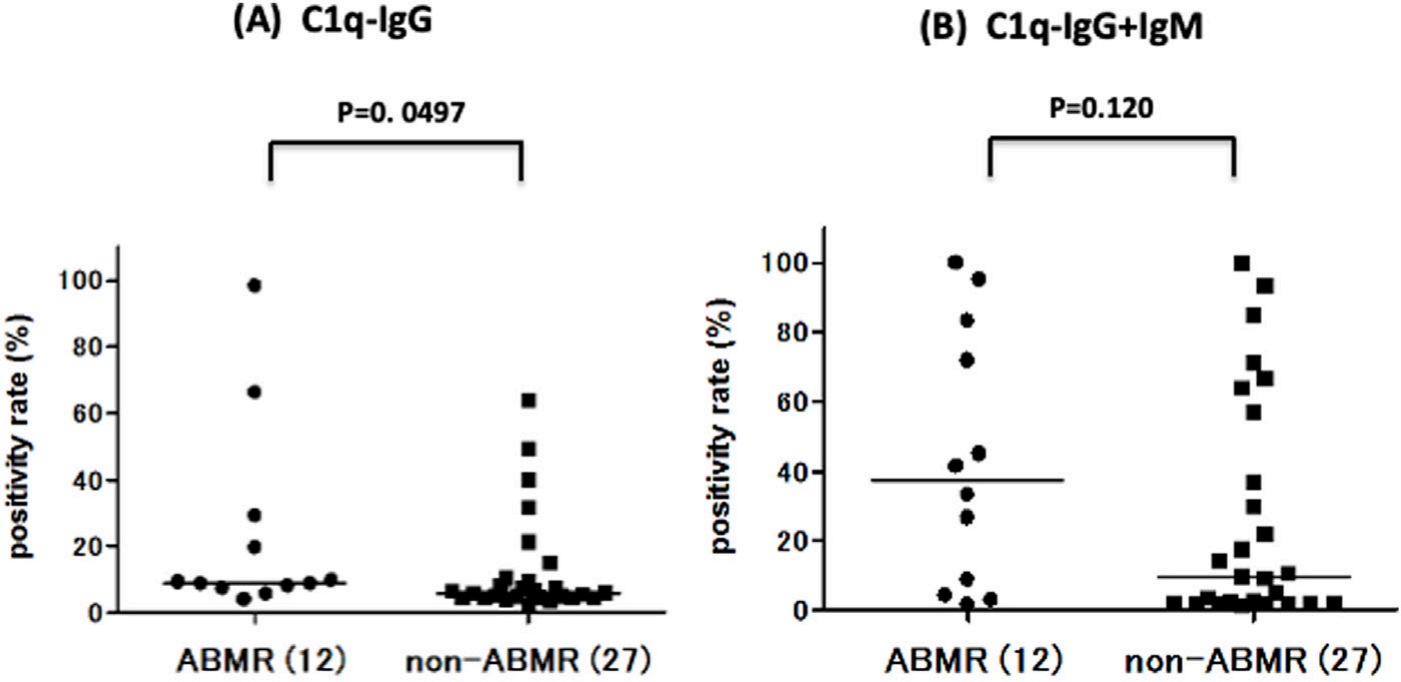
Comparison of anti-A complement C1q titer between ABMR and non-ABMR groups. There was a significant difference between groups in C1q, after removing the influence of IgM binding by DTT, there was a significant difference between groups in anti-A C1q−IgG positive rate (%) [anti-A: *p* = 0.049; **(A)**] in C1q−IgG+IgM positive rate (%) [anti-A: *p* = 0.120; **(B)**].

C1q binding ability was measured under C1q−IgG and C1q−IgG+IgM (Figure 5). The positivity rates of C1q binding to anti-A antibody were compared between ABMR and non-ABMR groups. C1q−IgG positivity rates were significantly higher in the ABMR group than in the non-ABMR group [DTT-treated C1q, 9.04% (25th–75th percentile, 7.63–26.7) vs. 5.93 (25th–75th percentile, 4.48–10.3), *p* = 0.049 in anti-A (Figure 5A; Table 2)], as were C1q−IgG+IgM positivity rates [DTT-non-treated C1q, 37.4% (25th–75th percentile, 5.48–80.5) vs. 9.70 (25th–75th percentile, 2.00–57.1), *p* = 0.120 in anti-A (Figure 5B; Table 2)]. The MFI cut-off values were calculated from ROC analysis (C1q−IgG: 7.47% AUC = 0.701, C1q: 26.6%, AUC = 0.659; Table 2).

## Discussion

ABO(H) antigens are oligosaccharides expressed as glycoproteins or glycolipids on cells and tissues, synthesized by glycosyltransferase from different precursor chains based on subtype-1,2,3,4 glycans in humans, depending on the type of cell or tissue [28]. Jeyakatanthan et al. reported differential subtype antigen expression between RBC and tissues or organs [15]. In this study, the quantitative analysis of A/B antigen in Kt, RBC, and Plt demonstrated that neither was associated with ABMR, despite the large inter-individual differences observed in Plt. Ogasawara et al. reported that 7% of Japanese had high A and B antigen expression on Plt [9], and Curtis et al. also found that 7% and 4% of Caucasians showed high A and B antigen expression on Plt, respectively [10]. However, our data did not show a positive correlation between high A/B antigen expression on Plt and ABMR.

The origin of anti-A/B antibodies is still controversial, but the natural antibodies appearing in the neonatal period (3–6 months) are IgMs [29, 30]. Although natural antibodies are usually produced in the absence of exogenous antigens, adult humans have anti-A/B antibodies of the IgG and IgA types produced by sensitization to food, bacteria and viruses which have similar antigens to those of A/B antigens [31]. ABO antigens are glycoprotein antigens, unlike HLA protein antigens. In general, protein antigens promote IgG1 and IgG3 production in B cells, after activation by T cells, whereas glycoprotein antigens mostly promote IgG2 and IgG4 production by B cells in the absence of T cells [32, 33]. The strength of complement activation varies by IgG subclass [18, 19]. IgG1 and IgG3 have a strong affinity for C1q, the first component of the complement pathway, and can thereby activate the complement [34]. Although IgG2 has a weaker complement activation ability than IgG1 and IgG3, the induction of complement activation depends on the density of antigen and antibody [19]. Therefore, high antibody titers of IgG2 can also activate the complement. It is also not yet clear which isotype (IgG or IgM) is more clinically important in ABO-I [35–37]. In the present study, we examined the total IgG/IgM, IgG subclass, and C1q binding ability to IgG/IgG+IgM ABO antibodies in the serum of patients undergoing pre-desensitization therapy. Higher IgG levels were more likely to be a risk factor for acute ABMR than IgM, but there was no significant difference between ABMR and non-ABMR groups. There was also a trend among IgG subclasses toward higher IgG1 and IgG2 levels being risk factors for ABMR, but there was no significant difference among subclasses between ABMR and non-ABMR groups. Comparatively, C1q binding ability (C1q-IgG) is likely to be a marker for ABMR, given the significant differences between ABMR and non-ABMR groups. The C1q binding ability to anti-A antibodies may reflect the density of IgG1 and IgG2 antibodies bound to ABO antigens. Schaub et al. reported that the C1q binding ability to HLA antibodies only reflects the density of bound antibodies and not the composition of IgG subclasses (IgG1-IgG4) [38].

The slightly worse graft engraftment rate of ABO-I compared to ABO-Id/C might be due to side effects such as infection and malignancy or cardiovascular disease [6]. Moreover, renal transplant recipients receiving RIT therapy are less likely to produce antibodies against SARS-Cov-2 [39]. Therefore, introduction of RIT-avoidance (free) protocol may be preferable and could be considered in a certain group [40, 41]. To safely implement such a protocol, we analyzed the association between the C1q binding ability and ABMR, and showed a possibility that C1q binding ability might be a useful marker for RIT avoidance (reduction).

This study has some limitations, including its cross-sectional design (one-point test) which does not allow analyzing changes over time; in addition, there was heterogeneity in immunosuppressive therapy. Nevertheless, this study has two strengths. First, we conducted analysis of antigen expression levels on donors. Second, a complement binding assay, used for detailed examination of HLA antibodies, could be applied to anti-A/B antibodies as well, even if a DTT treatment was necessary to remove the influence of anti-A/B IgM antibodies.

In conclusion, although the amount of A/B antigen in donors cannot explain ABMR in ABO-I, C1q binding ability could be a risk factor for ABMR. Further prospective studies are needed to justify a reduction in desensitization therapy.

## Data Availability

The raw data supporting the conclusions of this article will be made available by the authors, without undue reservation.

## Supplementary Material

Supplementary Data 1 Measuring of the DAB stain area of A/B antigens using the image analysis software Tissuemorph DP. The anti-A/B antibodies were diluted (dilution magnification ranged from ×25 to ×800) and compared in each sample with the highest DAB/ROI value.

Supplementary Data 2 Calculation of the MFI cut-off values of anti-A IgG, IgM, IgG subclasses antibody and C1q-binding-ability by ROC analysis.

Supplementary Data 3 Correlation between hemagglutinin titers and MFI of anti-A antibody IgG and IgM.

## Abbreviations

ABO-I, ABO blood group incompatible kidney transplantation; ABO-Id/C, ABO blood group identical or compatible kidney transplantation; ABMR, Antibody-mediated rejection; AR, Acute rejection; AUC, Area under the ROC curve; CV, Coefficient of variation; DSA, Donor specific HLA antibody; EC, Endothelial cells; IHT, Isohemagglutinin titer; IST, Immunosuppressive therapy; Kt, Kidney tissues; MFI, Mean fluorescence intensity; NoR/S, Neither rituximab nor splenectomy; Plt, Platelets; RBC, Red blood cells; RIT, Rituximab; ROC, Receiver operating characteristic; ROI, Region of interest; SPX, Splenectomy.
