# Supplementary material for: C1q Binding Ability for Prior Risk Assessment of Acute Antibody-Mediated Rejection in ABO-Incompatible Kidney Transplantation
Source: Transpl Int. 2024 Oct 15;37:13407. doi: 10.3389/ti.2024.13407 (PMC11518703; doi:10.3389/ti.2024.13407)
Supplement: Supplementary file 1 [file Presentation1.pptx]

## Slide 1
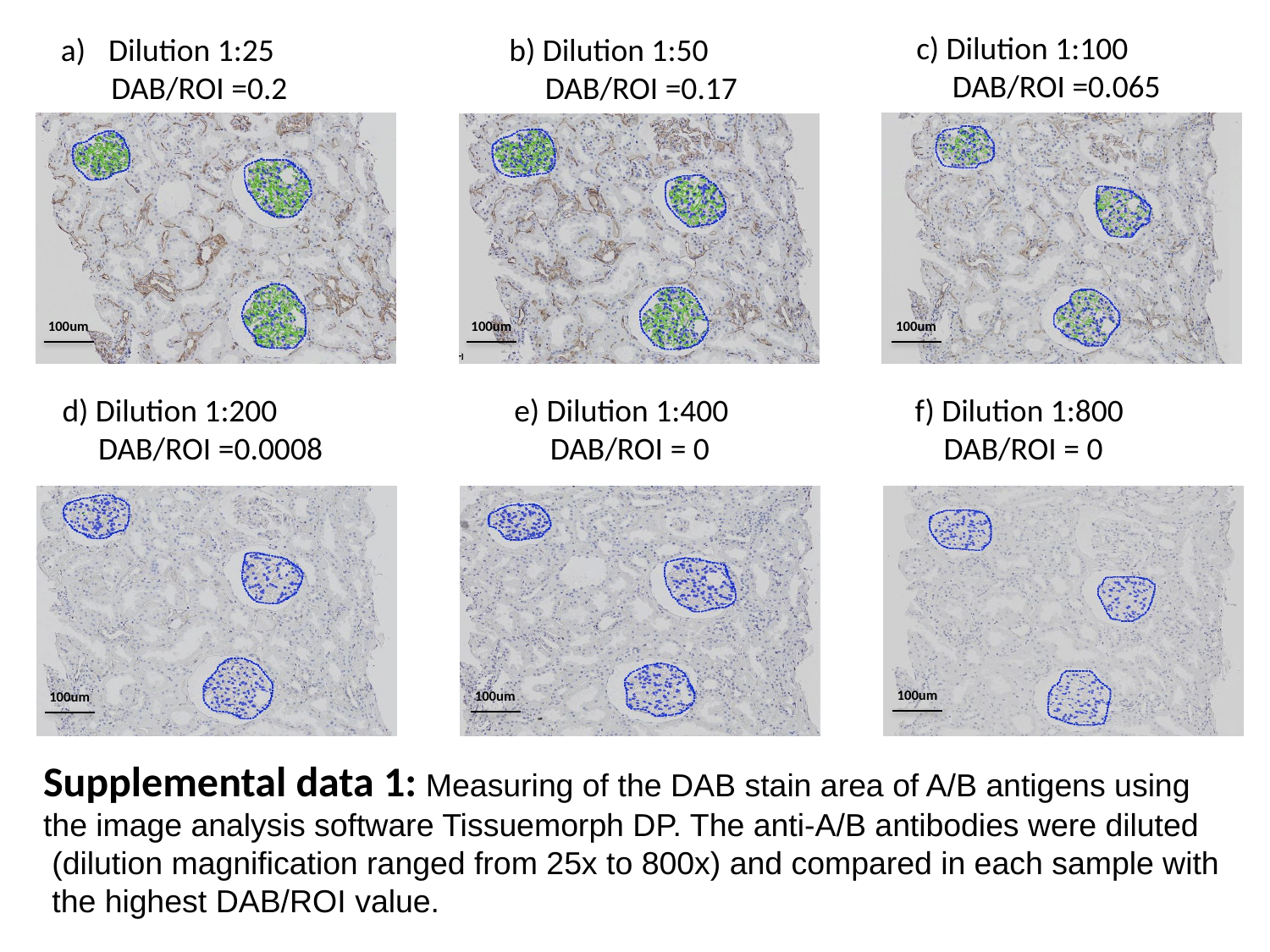

c) Dilution 1:100
 DAB/ROI =0.065
Dilution 1:25
 DAB/ROI =0.2
b) Dilution 1:50
 DAB/ROI =0.17
100um
100um
100um
d) Dilution 1:200
 DAB/ROI =0.0008
e) Dilution 1:400
 DAB/ROI = 0
f) Dilution 1:800
 DAB/ROI = 0
100um
100um
100um
Supplemental data 1: Measuring of the DAB stain area of A/B antigens using
the image analysis software Tissuemorph DP. The anti-A/B antibodies were diluted
 (dilution magnification ranged from 25x to 800x) and compared in each sample with
 the highest DAB/ROI value.

## Slide 2
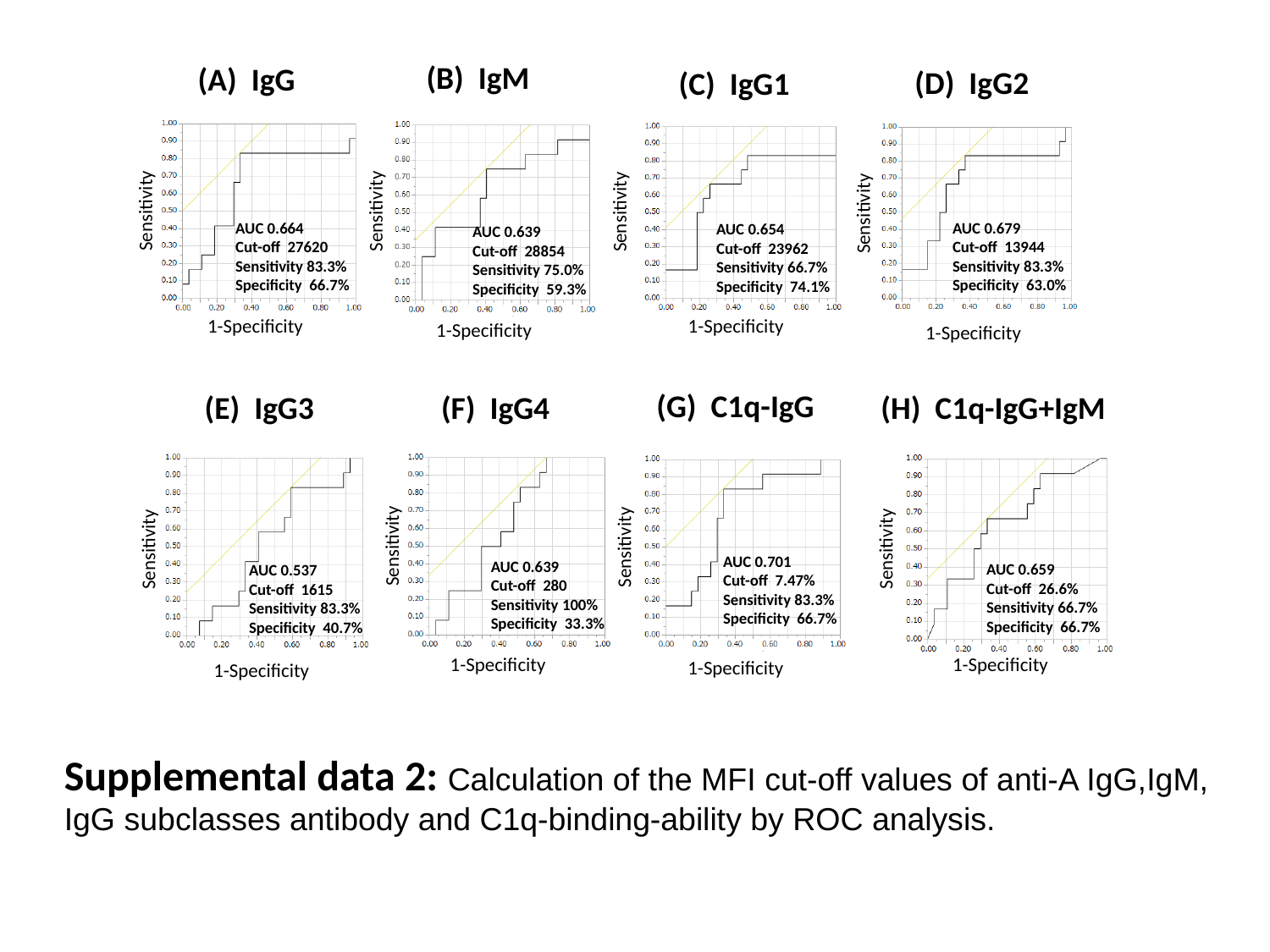

(B) IgM
 (A) IgG
 (D) IgG2
 (C) IgG1
Sensitivity
Sensitivity
Sensitivity
Sensitivity
AUC 0.679
Cut-off 13944
Sensitivity 83.3%
Specificity 63.0%
AUC 0.664
Cut-off 27620
Sensitivity 83.3%
Specificity 66.7%
AUC 0.654
Cut-off 23962
Sensitivity 66.7%
Specificity 74.1%
AUC 0.639
Cut-off 28854
Sensitivity 75.0%
Specificity 59.3%
1-Specificity
1-Specificity
1-Specificity
1-Specificity
 (G) C1q-IgG
 (H) C1q-IgG+IgM
 (E) IgG3
 (F) IgG4
Sensitivity
Sensitivity
Sensitivity
Sensitivity
AUC 0.701
Cut-off 7.47%
Sensitivity 83.3%
Specificity 66.7%
AUC 0.639
Cut-off 280
Sensitivity 100%
Specificity 33.3%
AUC 0.659
Cut-off 26.6%
Sensitivity 66.7%
Specificity 66.7%
AUC 0.537
Cut-off 1615
Sensitivity 83.3%
Specificity 40.7%
1-Specificity
1-Specificity
1-Specificity
1-Specificity
Supplemental data 2: Calculation of the MFI cut-off values of anti-A IgG,IgM,
IgG subclasses antibody and C1q-binding-ability by ROC analysis.

## Slide 3
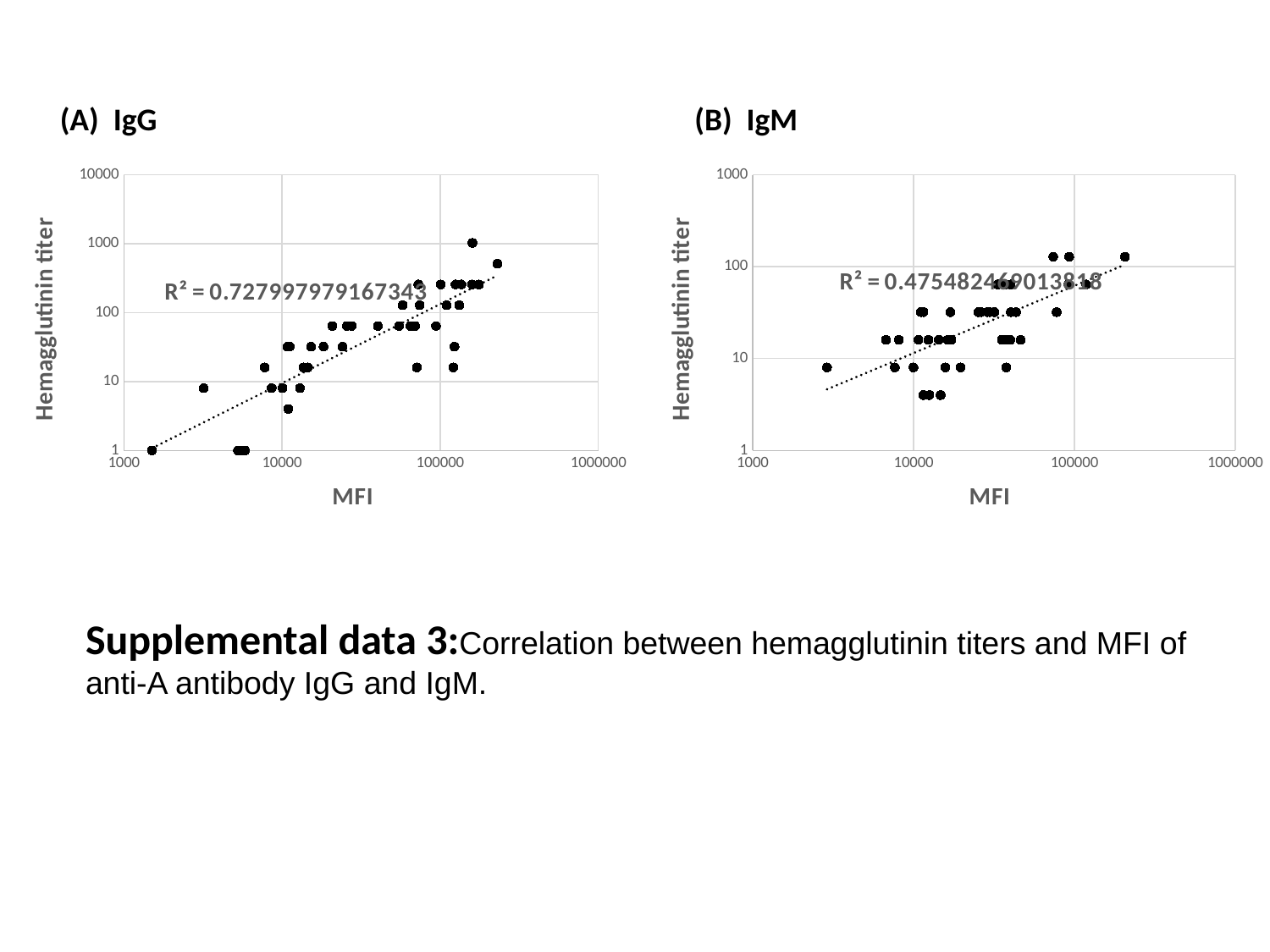

(A) IgG
 (B) IgM
### Chart
| Category | IgG 凝集反応（DC) |
|---|---|
### Chart
| Category | IgM 凝集反応(NS) |
|---|---|Supplemental data 3:Correlation between hemagglutinin titers and MFI of
anti-A antibody IgG and IgM.
